# Ebola epidemic - the Nigerian experience

**DOI:** 10.11694/pamj.supp.2015.22.1.6625

**Published:** 2015-10-11

**Authors:** Ugochukwu Uchenna Onyeonoro, Uchechi Chukwudike Ekpemiro, Chuku Abali, Huldah Ijeoma Nwokeukwu

**Affiliations:** 1 Department of Community Medicine, Federal Medical Center, Umuahia, Abia State, Nigeria; 2 Department of Surgery, Federal Medical Center, Umuahia, Abia State, Nigeria; 3 Department of Ophthalmology, Federal Medical Center, Umuahia, Abia State, Nigeria

**Keywords:** Ebola, hygiene, impact, public health, Nigeria

## Abstract

The current West African ebola epidemic has been described as the most unprecedented in the history of the disease. Nigeria reported its first case of the disease in July, 2014, at the end of the epidemic 20 people were infected and eight of them died. The epidemic resulted in increased knowledge of the disease as well as some misconceptions, increase in household and community hygiene practice and change in social interaction between affected individuals and the community. Prompt response by the government, with the support of international partners and proactive engagement of public health measures resulted in the rapid control of the epidemic; an experience the country hopes to leverage upon in subsequent epidemics.

## Introduction

The current Ebola epidemic in West Africa has been described as the largest, most severe and most complex outbreak since the first case of the disease was reported in 1976 [1]. Recent World Health Organisation (WHO) situation report shows a total 27, 401 cases and 10,194 deaths since the epidemic began [2]. Although there has been significant reduction in the epidemic, there is need to x-ray opportunities presented by the epidemic in the context of global health, and that is exactly the essence of this write-up. While the global health community were presented with the challenge of coping with an Ebola Viral Disease (EVD) epidemic of such magnitude for the first time, it also afforded them the opportunity of exploring and discovering new diagnostic tools, treatment options, and trial of new potential vaccines as well as demonstrating the efficacy of public health interventions. Presently there is no known cure for the disease neither an approved vaccine that protects against the disease; hence prevention and control of the disease are limited to public health measures. The origin of the current outbreak has been traced to a 2-year old boy in the remote Guinean village of Meliandou who fell ill with a mysterious illness characterized by fever, black stools, and vomiting and died 2 days later in late December, 2013 [3]. Since then the disease has spread affecting a total of nine (9) countries. Most significantly affected were three West African countries Liberia, Guinea and Sierra Leone, while others include Senegal, Nigeria and Mali (less significantly affected), United States, Spain and United Kingdom [4]. The Nigerian index case was an imported one involving a 40-year old man, who arrived on board an aircraft from Liberia. The epidemic lasted for a total of 92 days from the day the first case was reported (20th July, 2014) till the day the country was declared Ebola free by WHO (20th October, 2014). At the end of the epidemic there were a total of 1424 contacts linked to the index case, 20 cases (19 confirmed and 1 probable) reported in 2 major cities in the country - Lagos and Port Harcourt, out of which 8 (7 confirmed and 1 probable) of them died, hence the estimated case fatality rate of EVD in Nigeria was 40.0% [5–7].

## Impact of Ebola epidemic

Although it is difficult to objectively quantify the impact of Ebola epidemic in the country, highlighted below are some effects of the disease on individuals, households and communities in the country. At individual and household levels, those affected by the disease are likely to suffer significant morbidity and death. Symptoms associated with EVD include fever, bleeding abnormalities and severe loss of fluid and electrolyte. Death often results from organ shut-down consequent upon severe dehydration, electrolyte imbalance, bleeding and shock. Socially, survivors and contacts of those infected with the disease and their relatives were highly stigmatised during and beyond the period of epidemic which impacted adversely on their social life, thereby making it difficult for them to relate with other members of the community [8]. Some of them were even suspended from work by their employers. Furthermore, at some point in the course of the epidemic, travellers from Nigeria were subjected to Ebola screening by some overseas countries. At the community level, Ebola disrupted the social life of the people, by limiting social gatherings and the use of handshake, which is a common form of greeting among Nigerians. However, religious activities were not significantly affected. Ebola not only changed the way people lived and interacted with each other, it also resulted in significant change in behaviour. Key among such changes was increase in hand hygiene practices at home and public places. Hand sanitizers and water/soap were placed in strategic locations in public and private institutions to promote hand hygiene practice, while Ebola screening measures were introduced in all border points as well as selected public places.

The social impact of the disease at the community level was aggravated by some misinformation spread via social media and text messages. Among which are that Garcinia Kola (Bitter Kola) can cure the disease. This assumption was sequel to the findings of a research conducted several years ago by a renowned Nigerian professor of pharmacy that the bitter kola can halt the replication of the organism [9], an assertion that he maintained while the epidemic was still on going. This led to increased consumption of the product, resulting in its scarcity. Another misinformation that went viral in the country during the epidemic was that Ebola is airborne and bathing with and drinking salt and hot water combination can cure or protect against Ebola. This rumour led to several people ingesting high concentration of salt solution, resulting in death and hospitalisation of 2 and 20 persons in Jos, Plateau State respectively [10]. Finally, evidence from severely affected countries suggests that the economic impact of Ebola can be far reaching. Although, the country did not suffer long-lasting epidemic, economic losses could arise from loss of household income, loss of employees of businesses and public institutions to deaths, illness and fear resulting in slowing down of economic activities [11, 12].

## Public health response and outcome-lessons learnt

**Figure 1:**
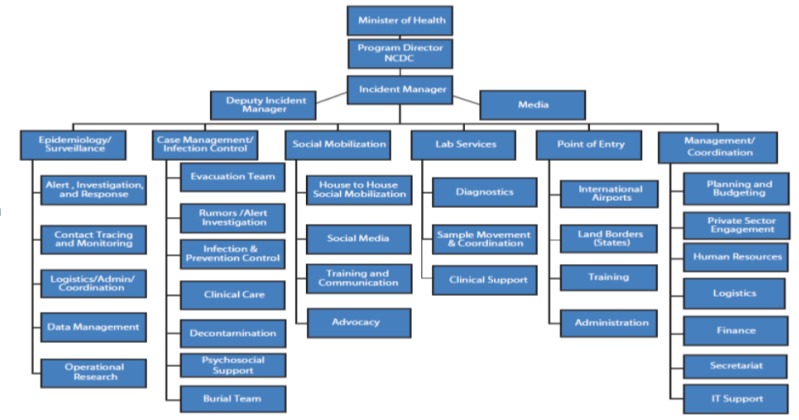
Organisational structure of the Ebola Response Incident Center Nigeria

In the words of former Nigerian Minister of Health “It's possible to control Ebola. It's possible to defeat Ebola. We've seen it here in Nigeria. If any cases emerge in the future, it will be considered-by international standards-a separate outbreak. If that happens, Nigeria will be ready and able to confront it exactly as we have done with this outbreak”. The country's response to the EVD epidemic has been found to be among the best public health practices globally. In recognition of the threat posed by the epidemic, the country commenced emergency preparedness efforts early with the assistance of the international partners which included capacity building. Consequently, upon confirmation of the first case in the country, a state of emergency was declared, allowing the Ministry of Health to establish Ebola Emergency Operations Center (EOC). Furthermore, the country leveraged on experience gained in the previous outbreak responses such as major lead poisoning response in 2010, and the recent experience with polio eradication and use of its national public institution like the Nigerian Center for Disease Control (NCDC)). Six response teams were developed within the EOC specific to Ebola response and they included 1) Epidemiology/Surveillance, 2) Case Management/Infection Control, 3) Social Mobilization, 4) Laboratory Services, 5) Point of Entry, and 6) Management/Coordination (Figure 1). The mandate of the EOC includes among others; case management of confirmed cases, contact tracing and surveillance, information management, coordination and social mobilisation including mobilising international support [13]. Key outcomes of the national response include among others; halting of the epidemic, increased awareness of the disease, increase hand hygiene practice both at household and community level, increased capacity of health care system and collaboration of local and international partners in epidemic control.

## Conclusion

Even though the country is free from the disease and the epidemic is slowing down in the west African sub-region, we hereby recommend the following; the country's emergency response plan should be maintained, and efforts should be made to strengthen it; high level of surveillance of the associated risk factors should be sustained including among the animal population; high level of hygiene and sanitary practices attained during the peak of the epidemic should be encouraged as part of the overall measures of protecting the health of the public, and subsequent responses should incorporate provision of psychological support and rehabilitation of individuals and families affected by the disease.
